# Recurrent Intracerebral Hemorrhage: Associations with Comorbidities and Medicine with Antithrombotic Effects

**DOI:** 10.1371/journal.pone.0166223

**Published:** 2016-11-10

**Authors:** Linnea Boegeskov Schmidt, Sanne Goertz, Jan Wohlfahrt, Mads Melbye, Tina Noergaard Munch

**Affiliations:** 1 Department of Epidemiology Research, Statens Serum Institut, Copenhagen, Denmark; 2 Department of Neurosurgery, Copenhagen University Hospital, Copenhagen, Denmark; "INSERM", FRANCE

## Abstract

**Background:**

Intracerebral hemorrhage (ICH) is a disease with high mortality and a substantial risk of recurrence. However, the recurrence risk is poorly documented and the knowledge of potential predictors for recurrence among co-morbidities and medicine with antithrombotic effect is limited.

**Objectives:**

1) To estimate the short- and long-term cumulative risks of recurrent intracerebral hemorrhage (ICH). 2) To investigate associations between typical comorbid diseases, surgical treatment, use of medicine with antithrombotic effects, including antithrombotic treatment (ATT), selective serotonin reuptake inhibitors (SSRI’s), and nonsteroidal anti-inflammatory drugs (NSAID’s) with recurrent ICH.

**Methods:**

The cohort consisted of all individuals diagnosed with a primary ICH in Denmark 1996–2011. Information on comorbidities, surgical treatment for the primary ICH, and the use of ATT, SSRI’s and NSAID’s was retrieved from the Danish national health registers. The cumulative recurrence risk of ICH was estimated using the Aalen-Johansen estimator, thus taking into account the competing risk of death. Associations with potential predictors of recurrent ICH were estimated as rate ratios (RR’s) using Poisson regression. Propensity score matching was used for the analyses of medicine with antithrombotic effects.

**Results:**

Among 15,270 individuals diagnosed with a primary ICH, 2,053 recurrences were recorded, resulting in cumulative recurrence risk of 8.9% after one year and 13.7% after five years. Surgical treatment and renal insufficiency were associated with increased recurrence risks (RR 1.64, 95% CI 1.39–1.93 and RR 1.72, 95% CI 1.34–2.17, respectively), whereas anti-hypertensive treatment was associated with a reduced risk (RR 0.82, 95% CI 0.74–0.91). We observed non-significant associations between the use of any of the investigated medicines with antithrombotic effect (ATT, SSRI’s, NSAID’s) and recurrent ICH.

**Conclusions:**

The substantial short-and long-term recurrence risks warrant aggressive management of hypertension following a primary ICH, particularly in patients treated surgically for the primary ICH, and patients with renal insufficiency.

## Introduction

According to few previous reports, between 1.3–7.4% of survivors of an intracerebral hemorrhage (ICH) experience recurrence within a year[1] and up to 18.8% experience recurrence within five years.[2] However, most studies have been limited in size and in particular estimates for the long-term risk are poorly documented.

Elderly age and hypertension remain the only consistently reported risk factors for recurrent ICH. Findings regarding other potential predictors among co-morbid diseases and use of antithrombotic treatment (ATT) are equivocal.[3–11] Previous studies are limited by size, loss to follow-up, and some by lack of information on confounders. The question whether the widely used selective serotonin reuptake inhibitors (SSRI’s) increase the recurrence risk of ICH has been raised in a recent meta-analysis reporting 42% higher risk of primary ICH.[12]

The virtually complete information from the nationwide Danish registers enabled us to estimate the short- and long-term cumulative recurrence risks of intracerebral hemorrhage, and to investigate potential predictors for recurrence among the typical comorbidities in this patient group. The detailed register information on prescription drugs in Denmark further enabled us to investigate the associations between ATT and SSRI’s and ICH recurrence.

## Materials and Methods

### Data sources

The Danish civil registration system records and continuously updates demographic information including vital status and emigration on every individual living in Denmark.[13] All individuals are assigned a personal identification number, allowing for cross linkage between all Danish health registers. In Denmark health care is free, easily accessible, and reporting to the national registers is mandatory.

Diagnoses for all hospital admittances since 1977, and since 1995 also emergency room visits and outpatient contacts, are recorded in the National Patient Register.[14] Diagnoses are registered according to the International Classification of Diseases. All ICD-codes used in this study are listed in S1 Table in the supplemental material.

The National Prescription Register has recorded information on subscribed medicine according to the Anatomical Therapeutic Chemical Classification System since 1995, including information on date of dispensing, number and strength of tablets.[15] The ATC-codes used in this study are listed in S1 Table in the supplemental material.

### Study cohort

From an unselected source population comprising every citizen living in Denmark between January 1996 and December 2011, the study cohort was defined as all patients aged 20 years or older admitted to a hospital with a first-time diagnosis of ICH. Individuals with an ICH diagnosis before 1996 and individuals diagnosed with a cerebral tumor, cerebral vascular malformation or any other type of intracranial bleeding prior to or during the admittance were excluded. Patients diagnosed with a cerebral infarction during the admittance were excluded, as the ICH was considered to represent a hemorrhagic transformation of the infarction (for relevant ICD-codes, see S1 Table in the supplemental material).

### Follow up and primary outcome

Entry to the study and follow up started seven days after discharge for a primary ICH. The hospitalization period for the primary event was defined as the first hospital stay and all subsequent admittances with less than seven intermittent days, thereby accounting for re-admittances for the same hemorrhage as well as transfer between hospitals. Patients were followed until the first of the following events: Recurrent ICH, end of study (December 31^st^ 2011), death, or emigration. Patients diagnosed with a different type of intracranial bleeding during follow up were censored from the date of that diagnosis.

Some re-admittances, typically within the first three months, may be related to complications of the primary ICH rather than recurrent ICH’s.[16] These patients may be assigned an ICH diagnosis, referring to the primary event. To account for this type of incorrect recording of ICH recurrence, patients re-admitted within the first 90 days of follow-up with one of the following diagnoses: pneumonia, sepsis, urinary tract infection, venous thromboembolism, seizure or other neurological symptoms, were censored during admittance.

### Potential predictors

Based on the literature, the potential predictors for recurrent ICH included age, gender, surgical treatment for the primary ICH, and the following diagnoses registered no later than during the hospitalization for the first bleeding: Hypertension, cerebral infarction, ischemic heart disease, atrial fibrillation, heart valve surgery, endocarditis, coagulopathy, diabetes mellitus, renal insufficiency, chronic liver disease, alcohol addiction, hypercholesterolemia, and pre-packed daily medication doses (PDMD). PDMD implies that a patient’s medicine is packed according to the date and time of intake by a pharmacist and supposedly represents patients of the heaviest disease burden. Information on comorbid diseases and surgical treatment was retrieved from the National Patient Register. Diabetes, hypertension, hypercholesterolemia and alcohol addiction are predominantly treated in primary care, and thus not always registered by diagnosis. For that reason, information on these diseases was based on dispensed medicine, identified in the National Prescription Register together with information on PDMD. The patients were considered exposed throughout follow-up.

### Antithrombotic treatment, SSRI’s and NSAID’s

Users of antithrombotic treatment (clopidogrel, acetylsalicylic acid, and warfarin), NSAID’s (nonsteroidal anti-inflammatory drugs), and SSRI’s after the primary ICH were matched 1:1 on propensity scores by nearest neighbor risk set matching. The propensity scores were estimated with Cox regression with filling of first prescription since the primary ICH as outcome, and with all predictors mentioned above and length of first hospital stay as covariates. Patients were thus considered users from first prescription and onwards. As NSAID’s are usually used for shorter time periods only, we ended follow-up at the end of the first consecutive period of medicine use. Filled prescriptions were identified in the Danish National Prescription Register.

### Statistical analyses

Overall cumulative risks of recurrent ICH since entry to the study were estimated using the Aalen-Johansen estimator, taking into account the competing risk of death. A log-linear Poisson regression model was used to estimate the rate ratios (RR’s) for exposed as compared to non-exposed cohort members. RR’s for potential predictors were adjusted for all other predictors listed in Table 1 (categorized as listed), current use of ATT, NSAID’s and SSRI’s (considered time-dependent in these analyses, see S1 Text), as well as calendar period (1996–2000, 2001–2005 and 2006–2011), time since admission for the primary ICH (0-<1 month, 1-<2 months, 3-<6 months, 6 months-<1 year and ≥ 1 year) and length of hospital stay for the primary ICH (quartiles (days) 1–8, 9–19, 20–45 and 46+). RR’s within the strata of surgically versus conservatively treated ICH’s and within strata of time since the primary ICH were estimated by adding an interaction term into the model, but the confounder effects were assumed to be similar in the two strata in these analyses. RR’s for medicine with antithrombotic effect were propensity score matched and adjusted for time since admittance for the primary ICH.

**Table 1:** Cumulative 1-year recurrence risks and rate ratios (RR) of recurrent intracerebral hemorrhage (ICH), according to comorbidities and other potential predictors in Denmark 1996–2011.

Potential predictors for recurrent ICH	No. of primary ICH	No. of recurrent ICH	Median follow-up time (days)	Cumulative 1-year recurrence risk in percent (95% CI^a^)		Adjusted RR^a^ (95% CI^b^)	
**All patients**	15270	2053					
**Age (years)**							
20–49	1473	222	1746	12.8	(11.0–14.4)	1	(Ref.)
50–69	5493	854	1397	10.4	(9.6–11.2)	1.09	(0.94–1.27)
70-	8304	977	784	7.2	(6.6–7.7)	1.09	(0.93–1.27)
						P-value = 0.50	
**Sex**							
Males	7987	1122	1067	9.1	(8.5–9.7)	1.07	(0.98–1.17)
Females	7283	931	971	8.6	(8.0–9.3)	1	(Ref.)
**Surgical treatment**							
Yes	669	173	1011	21.2	(18.1–24.3)	1.64	(1.39–1.93)
No	14601	1880	1020	8.3	(7.8–8.7)	1	(Ref.)
**Antihypertensive treatment**							
Yes	5854	636	839	7.5	(6.8–8.2)	0.82	(0.74–0.91)
No	9416	1417	1152	9.7	(9.1–10.3)	1	(Ref.)
**Previous cerebral infarction**							
Yes	2149	269	821	8.6	(7.4–9.8)	0.90	(0.79–1.02)
No	13121	1784	1049	8.9	(8.4–9.4)	1	(Ref.)
**Ischemic heart disease**							
Yes	2428	271	761	8.2	(7.1–9.3)	0.92	(0.80–1.05)
No	12842	1782	1076	9.0	(8.5–9.5)	1	(Ref.)
**Atrial fibrillation**							
Yes	1883	174	625	6.6	(5.5–7.7)	0.80	(0.68–0.94)
No	13387	1879	1095	9.2	(8.7–9.7)	1	(ref.)
**Heart valve surgery**							
Yes	155	20	950	11.8	(6.6–16.9)	1.03	(0.63–1.59)
No	15155	2033	1021	8.8	(8.4–9.3)	1	(Ref.)
**Endocarditis**							
Yes	31	4	905	9.8	(0–20.5)	1.02	(0.31–2.43)
No	15239	2049	1020	8.9	(8.4–9.3)	1	(Ref.)
**Coagulopathy**							
Yes	132	13	586	9.2	(4.3–14.2)	0.75	(0.41–1.25)
No	15138	2040	1025	8.9	(8.4–9.3)	1	(Ref.)
**Diabetes mellitus**							
Yes	711	78	667	8.9	(6.8–11.0)	1.00	(0.79–1.26)
No	14559	1975	1042	8.9	(8.4–9.3)	1	(Ref.)
**Renal insufficiency**							
Yes	394	72	417	16.1	(12.4–19.7)	1.72	(1.34–2.17)
No	14876	1981	1040	8.7	(8.2–9.1)	1	(Ref.)
**Chronic liver disease**							
Yes	466	57	661	9.8	(7.1–12.6)	1.03	(0.77–1.36)
No	14804	1996	1032	8.8	(8.4–9.3)	1	(Ref.)
**Alcohol addiction**							
Yes	1427	171	892	9.0	(7.5–10.4)	0.90	(0.75–1.07)
No	13843	1882	1032	8.9	(8.4–9.3)	1	(Ref.)
**Statins**							
Yes	2258	243	824	8.2	(7.1–9.4)	1.01	(0.86–1.17)
No	13012	1810	1067	9.0	(8.5–9.5)	1	(Ref.)
**PDMD**^c^							
Yes	307	27	345	7.7	(4.7–10.7)	1.22	(0.81–1.76)
No	14963	2026	1039	8.9	(8.4–9.3)	1	(Ref.)

^a^ Adjusted for age, sex, calendar period, time since admittance, length of hospital stay for the first bleeding, all other listed potential predictors for recurrent ICH, and current use of antithrombotic therapy, NSAID’s or SSRI’s.

^b^ 95% confidence interval

^c^ PDMD: Pre-packaged Daily Medication Doses

For all RR’s, confidence intervals were 95% likelihood ratio intervals, and all tests were likelihood ratio tests with a significance level of 0.05.

### Ethical considerations

The study was approved by the Danish Data Protection Agency. Ethical approval is not required for register-based studies in Denmark.

## Results

**Fig 1:**
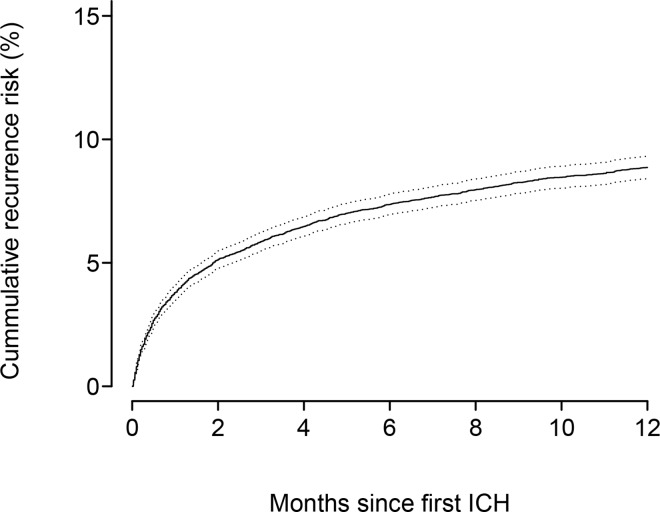
The cumulative risk in percent of recurrent intracerebral hemorrhage (ICH) up to one year after the first ICH.

A total of 15,270 individuals were diagnosed with a primary ICH, of which 2,053 individuals experienced a recurrence during follow-up. Fig 1 shows the cumulative recurrence risk in percent up to one year after the primary ICH. At four weeks after the primary ICH, the cumulative recurrence risk was 3.67% (95% CI: 3.37–3.97%), increasing to 5.86% (95% CI: 5.48–6.23%) at 90 days, 8.86% (95% CI: 8.41–9.31%) at one year, continuously increasing to a five-year cumulative recurrence risk of 13.72% (95% CI: 13.15–14.29%).

### Comorbidities and recurrence risk

The recurrence risks of ICH, estimated as rate ratios (RR’s) and the 1-year cumulative risks, according to selected comorbidities and other potential predictors are presented in Table 1. A significantly increased recurrence risk was observed for patients treated surgically for the primary ICH (RR 1.64 95% CI: 1.39–1.93), compared to conservatively treated patients. The 1-year cumulative recurrence risks were 21.2% for surgically treated patients compared to 8.3% for conservatively treated patients.

Patients with renal insufficiency had a significantly increased recurrence risk (RR 1.72, 95% CI: 1.34–2.17) with a 1-year cumulative recurrence risk of 16.1% compared to 8.7% for other cohort members.

Antihypertensive treatment was associated with a significantly reduced recurrence risk (RR 0.82, 95% CI: 0.74–0.91). The 1-year cumulative recurrence risk for patients treated for hypertension was 7.5% compared to 9.7% for others.

Atrial fibrillation was associated with a reduced recurrence risk, also evident before adjustment for ATT (fully adjusted RR 0.80, 95% CI: 0.68–0.94; RR adjusted for other predictors only 0.81, 95% CI: 0.69–0.96). The 1-year cumulative recurrence risk was 6.6% for patients with atrial fibrillation, compared to 9.2% for others.

Because the overall recurrence risk of ICH is most pronounced within the first year after the primary ICH, we estimated rate ratios for each of the significant predictors within and after one year. Results are presented in Table 2. Surgical treatment for the primary ICH (RR <1 year: 2.00, 95% CI 1.64–2.41; RR ≥1 year 1.09, 95% CI: 0.79–1.47 (p = 0.0007)) and renal insufficiency (RR <1 year 2.07, 95% CI: 1.58–2.66; RR ≥1 year 0.86, 95% CI: 0.43–1.51 (p = 0.005)) were associated with significantly higher recurrence risks within the first year after the primary ICH compared to later. Patients aged 70 years or older at the time of the primary ICH had the lowest recurrence risk within the first year and the highest recurrence risk after the first year, compared to the youngest patient group (RR_<1 year, 70- vs. 20–49 years_ 0.71, 95% CI: 0.59–0.85 and RR_≥1 year, 70- vs. 20–49 years_ 2.52, 95% CI: 1.89–3.43 (p < 0.0001)).

**Table 2:** Rate ratio (RR) of recurrent intracerebral hemorrhage (ICH) within and after the first year of the primary ICH, according to comorbidities and other potential predictors in Denmark 1996–2011.

Potential predictors for recurrent ICH	<1 year			≥1 year			P-value^c^
	Events	Adjusted RR^a^	(95% CI^b^)	Events	Adjusted RR^a^	(95% CI^b^)	
**Age (years)**							
20–49	171	1	(Ref.)	51	1	(Ref.)	
50–69	539	0.87	(0.73–1.04)	315	1.83	(1.37–2.49)	<0.0001
70-	569	0.71	(0.59–0.85)	408	2.52	(1.89–3.43)	
**Sex**							
Males	684	1.03	(0.92–1.15)	438	1.14	(0.99–1.32)	0.27
Females	595	1	(Ref.)	336	1	(Ref.)	
**Surgical treatment**							
Yes	130	2.00	(1.64–2.41)	43	1.09	(0.79–1.47)	0.0007
No	1149	1	(Ref.)	731	1	(Ref.)	
**Antihypertensive treatment**							
Yes	414	0.85	(0.75–0.96)	222	0.78	(0.66–0.91)	0.36
No	865	1	(Ref.)	552	1	(Ref.)	
**Previous cerebral infarction**							
Yes	174	0.84	(0.71–0.98)	95	1.03	(0.82–1.27)	0.14
No	1105	1	(Ref.)	679	1	(Ref.)	
**Ischemic heart disease**							
Yes	186	0.95	(0.80–1.11)	85	0.87	(0.69–1.09)	0.55
No	1093	1	(Ref.)	689	1	(Ref.)	
**Atrial fibrillation**							
Yes	119	0.78	(0.64–0.94)	55	0.85	(0.63–1.11)	0.64
No	1160	1	(Ref.)	719	1	(Ref.)	
**Heart valve surgery**							
Yes	18	1.46	(0.87–2.28)	2	0.28	(0.05–0.89)	0.007
No	1261	1	(Ref.)	772	1	(Ref.)	
**Endocarditis**							
Yes	3	1.12	(0.27–2.96)	1	0.81	(0.05–3.63)	0.78
No	1276	1	(Ref.)	773	1	(Ref.)	
**Coagulopathy**							
Yes	11	0.92	(0.47–1.58)	2	0.38	(0.06–1.19)	0.21
No	1268	1	(Ref.)	772	1	(Ref.)	
**Diabetes mellitus**							
Yes	60	1.14	(0.86–1.47)	18	0.72	(0.44–1.12)	0.09
No	1219	1	(Ref.)	756	1	(Ref.)	
**Renal insufficiency**							
Yes	62	2.07	(1.58–2.66)	10	0.86	(0.43–1.51)	0.005
No	1217	1	(Ref.)	764	1	(Ref.)	
**Chronic liver disease**							
Yes	41	1.11	(0.79–1.53)	16	0.87	(0.51–1.40)	0.41
No	1238	1	(Ref.)	758	1	(Ref.)	
**Alcohol addiction**							
Yes	117	0.96	(0.78–1.17)	54	0.80	(0.59–1.05)	0.28
No	1162	1	(Ref.)	720	1	(Ref.)	
**Statins**							
Yes	170	1.05	(0.88–1.24)	73	0.93	(0.72–1.18)	0.42
No	1109	1	(Ref.)	701	1	(Ref.)	
**PDMD**^d^							
Yes	23	1.38	(0.88–2.06)	4	0.72	(0.22–1.69)	0.20
No	1256	1	(Ref.)	770	1	(Ref.)	

^a^ Rate ratios adjusted for age, sex, calendar period, time since admittance for the first ICH, length of hospital stay for the first bleeding, potential predictors listed in Table 1 and current use of antithrombotic treatment, NSAID’s and SSRI’s.

^b^ 95% confidence interval

^c^ P-value for homogeneity test for no difference between the RR’s within one year and one or more years following the first ICH.

^d^ PDMD: Pre-packaged Daily Medication Doses

The ICH’s found eligible for surgical treatment are usually selected according to size and location of the ICH, etiology, and patient age. Stratified analyses were conducted to enlighten potential differences in how the predictors affect the recurrence risk in surgically- versus conservatively treated patients. As shown in S2 Table, the results revealed no significant differences.

### Medicine with antithrombotic effect and recurrence risks

Rate ratios for recurrence according to antithrombotic treatment, selective serotonin reuptake inhibitors and nonsteroidal anti-inflammatory drugs are presented in Table 3.

**Table 3:** Rate ratios (RR)^a^ of recurrent intracerebral hemorrhage (ICH) according to the use of antithrombotic medicine, selective serotonin reuptake inhibitors, and nonsteroidal anti-inflammatory drugs in Denmark 1996–2011.

	Users		Non-users		RR (95% CI^b^)
	Person-years	Events	Person-years	Events	
Antithrombotic treatment					
*Current user at first ICH*					
Any ATT	2970	81	1360	58	0.76 (0.54–1.08)
ASA	2895	82	1504	55	0.89 (0.63–1.26)
Clopidogrel	361	7	230	12	0.40 (0.15–1.00)
Warfarin	656	7	405	10	0.55 (0.20–1.43)
*Never user at first ICH*					
Any ATT	5714	112	3494	92	0.80 (0.61–1.06)
ASA	5064	97	3291	67	0.99 (0.73–1.36)
Clopidogrel	505	10	373	13	0.56 (0.24–1.28)
Warfarin	803	17	554	11	1.09 (0.52–2.40)
**SSRI**	14732	379	11298	320	0.95 (0.82–1.10)
**NSAID**^**d**^	245	19	159	17	0.79 (0.41–1.54)

^a^ Matched for propensity score including all variables listed in Table 1 and length of hospital stay for the first bleeding. Rate Ratios were adjusted for time since admittance for the first ICH.

^b^ 95% confidence interval.

#### Antithrombotic treatment (ATT)

To enlighten potential differences between the recurrence risks of patients receiving ATT at the time of the primary ICH and the patients only initiating ATT after the primary ICH, separate analyses were conducted for these two groups, as well as stratification for each type of ATT (warfarin, clopidogrel and acetylsalicylic acid).

Among the 2,252 cohort members using antithrombotic medicine at the time of the primary ICH, 1101 were also users of ATT after the primary ICH, to which we were able to match 887 non-users. According to the results presented in Table 3, resumption of ATT after a primary ATT-related ICH was not significantly associated with recurrent ICH (RR 0.76, 95% CI: 0.54–1.08).

For the 9,492 cohort members who had never received ATT before or at the time of their primary ICH, 1358 used ATT after the primary ICH, of which 1357 were matched to a non-user. For these patients, the subsequent use of ATT was not significantly associated with recurrent ICH (RR 0.80, 95% CI: 0.61–1.06).

#### Selective serotonin reuptake inhibitors

We identified 3,998 cohort members using SSRI after the primary ICH, of which 3,952 were matched with non-users. SSRI treatment was not found to be associated with ICH recurrence (RR 0.95, 95% CI: 0.82–1.10).

#### Nonsteroidal anti-inflammatory drugs

3,124 cohort members were prescribed NSAID’s after the primary ICH and were matched 1:1 with non-users. We did not observe an increased recurrence risk during NSAID treatment (RR 0.79, 95% CI 0.41–1.54).

## Discussion

In this nationwide cohort study, we found a cumulative recurrence risk of 8.9% after one year and 13.7% after five years. Renal insufficiency at the time of the primary ICH predicted a 72% higher risk of recurrence than others, and surgical treatment for the primary ICH predicted an increased recurrence risk of 64% compared to conservatively treated patients. Antihypertensive treatment decreased the recurrence risk of ICH with 18%. The use of antithrombotic medicine (ATT), SSRI’s or NSAID’s after a primary ICH was not associated with ICH recurrence.

The 1-year cumulative recurrence risk of 8.9% is somewhat higher than the 1.8–7.4% Poon et al. reported in a recent review.[1] The nationwide register-based design of our study minimized the loss to follow up often seen in other studies, which implies a very high detection of recurrences. Since we censored re-admittances within the first three months, likely to represent complications of the primary ICH rather than true ICH recurrences, we believe the reported 1-year cumulative recurrence risk is a realistic estimate. Regarding the long-term recurrence risk, we observed a 5-year recurrence risk of 13.7%, which is in line with the few other studies with long-term follow up, reporting cumulative risks ranging from 11 to 18.8%.[2,3,5,11]

The increased recurrence risk observed in surgically treated patients was restricted to the first year, where surgical treatment more than doubled the risk of recurrence (Table 2). While the group of surgically treated ICH’s mostly consist of surgically accessible, collected hypertensive bleedings of a certain volume[17], the conservatively treated ICH’s have more different etiologies including cerebral amyloid angiopathy, small-volume hypertensive bleedings, substance abuse, and cerebral venous outflow disorders. Intuitively, one would think that the conservatively treated group is at least as likely to recur as surgically treated ICH’s. We speculate that the inflammatory response and traumatic brain edema following surgery may increase the risk of re-bleeding, but the increased risk in surgically treated patients remains unexplained.

Although the association between renal disease and recurrence of ICH has not been investigated previously, our finding of a particularly high risk for these patients is in line with studies reporting an increased risk of primary ICH with decreasing GFR and albuminuria.[18–20] Our finding supports particular attention to measures of blood coagulation following an ICH, in patients with renal insufficiency.

The reduced recurrence risk observed for patients using antihypertensive treatment likely reflects that a relatively large proportion of the population has undetected/untreated hypertension. Huhtakangas et al. reported a similar finding in a smaller cohort study.[4] These findings improve the evidence for the widely used aggressive blood pressure monitoring and management in the prevention of ICH recurrence.

We interpret the reduced recurrence risk in patients with a previous diagnosis atrial fibrillation to rely on the thrombotic nature of this disease, apparently outweighing the risk of intracerebral hemorrhage. Interestingly, this association was significant both before and after adjustment for antithrombotic treatment. In line with the result for atrial fibrillation, none of the investigated risk factors for atherosclerosis or other thromboembolic diseases were found to increase the recurrence risk.

We found no association between the use of ATT and ICH recurrence, neither in the group of patients who were using ATT at the time of the primary ICH, nor for the patients who had never been treated with ATT before the primary ICH. Most of the patients treated with ATT in our cohort used ASA. In line with our results, previous studies have not found any association with use of ASA and recurrent ICH, with the exception of one small study, only investigating recurrence of deep ICH’s in patients with cerebral amyloid angiopathy. [7,21–23] Importantly, the decision to prescribe ATT after an ICH must be based on a balanced estimate of the highly individual risk of thrombotic events versus the risk of recurrence. A study of 417 survivors of ICH, in which no increased recurrence rate was observed among the 120 patients starting antiplatelet therapy subsequently, the risk of ischemic events was three times higher in the group of patients who did not receive antiplatelet therapy.[7]

For warfarin therapy, Yung et al. investigated 284 patients with a warfarin-associated primary ICH, and found that resumption of warfarin was not associated with ICH recurrence,[11] which was confirmed by Kuramatsu et al. [9] We did not find a significantly increased recurrence risk within the strata of warfarin treatment, but the scarce number of cases really prohibits any conclusions. The low number of warfarin users may rely on a less restrictive attitude towards prescribing ASA rather than warfarin after an ICH.[11]

We observed no association between SSRI’s and recurrent ICH, which is a novel finding, as previous studies have only investigated the association with primary ICH. Hackam et al. reported a rate ratio of 1.42 (95% CI: 1.23–1.65), corresponding to an absolute risk of one additional ICH for every 10.000 patients using SSRI’s each year.[12] Another meta-analysis found an increase in hemorrhagic strokes with use of SSRI’s, but pointed out that depression itself may increase the risk of strokes.[24] However, based on the low absolute risk of primary ICH in previous studies[25] and the lack of association with recurrent ICH in this study, we find no evidence for hesitation in treating depression with SSRI’s after an ICH. Given the wide and increasing use of SSRI’s in the treatment of stroke-related depression, this finding is important. Especially as depression after a stroke is both common and has a considerable negative impact on functional outcome.[26] Furthermore, the non-significant association between use of NSAID’s and recurrent ICH is in line with previous findings.[27]

This population-based cohort study is, to our knowledge, the most comprehensive study estimating the short- and long-term cumulative recurrence risk of ICH as well as investigating a wide range of potential predictors of recurrence. The unique information sources provided by the Danish national registers ensured minimal selection bias and close to complete follow-up. Our access to information on actual medicine retrieval further strengthens the study by eliminating recall bias and any information bias caused by lack of compliance. Registration of hospital diagnoses in the Danish National Patient Register is mandatory and validation studies have found positive predictive values ranging from 92–100% for the diagnoses of comorbid diseases used in this study.[28–30] Unfortunately, we were not able to find any recent, reliable validation studies on the ICH diagnosis. The two existing studies include very few patients diagnosed either before, or during the very first years of follow-up. Since the interpretation of hemorrhagic findings on CAT scans obviously improved as CAT scans became very common and easily accessible in all hospitals from the late nineties and forward, we believe that the validity of the diagnosis improved significantly over the years. All analyses were adjusted for time period in order to counteract a potential effect on ICH diagnosis. Furthermore, we excluded individuals with more than one intracranial bleeding diagnosis. We may have missed fatal recurrent ICH’s, where death occurred prior to hospital admittance, which could lead to underestimation of the recurrence risk.

The register-based design of the study limited the availability of certain patient data, including clinical features of the primary ICH such as neurological status, hematoma size and anatomical localization. Although hematoma size and level of consciousness on admission for the primary ICH are associated with increased mortality and worsened long-term outcome, no studies have found these factors to be associated with recurrent ICH[1,4]. Previous studies have found that lobar localization, often seen in patients with cerebral amyloid angiopathy (CAA), is associated with recurrence.[8,19,31] The diagnosis CAA is not consistently registered, thus we were not able to investigate or adjust for it. As CAA is associated with elderly age, the missing information could potentially confound the results, and partially explain the high recurrence risk seen with elderly age after one year. Furthermore, we did not have information on lifestyle variables such as obesity and smoking, but the effects on the risk of ICH has been shown to mainly be mediated through diseases such as diabetes and hypertension, which were included in the analyses.[1,32,33]

Because ICH is a relative contraindication for ATT and no specific guidelines for restarting the medicine exist, the treating physician makes this decision for each individual patient. Thus, patients receiving ATT may have stronger indications for ATT (e.g. very high thrombotic risk), fewer risk factors for ICH or a less severe primary ICH, than the patients who are not started on ATT. Therefore, our results could be prone to confounding by indication.[34] However, given the wide access to other diagnoses and medicine use of the cohort members, we were able to include a wide range of potential confounders in the propensity score model for use of ATT.

In conclusion, we observed a pronounced short- and long-term recurrence risk of ICH, which is further increased after surgical treatment and for patients with renal insufficiency. In this by far largest cohort study of medicine with antithrombotic effect and recurrent ICH, we found no increased risk for users of ATT or NSAID’s after a primary ICH. Notably, we found no evidence of an increased risk for users of SSRI’s, an importing finding, since depression is commonly seen in patients who have suffered and ICH.

## Supporting Information

S1 Table Disease diagnoses and types of medicine, according to the International Classification of Diseases (ICD) and the Anatomical Therapeutic Chemical (ATC) Classification System. (DOCX)

S2 Table Rate ratio (RR) of recurrent intracerebral hemorrhage (ICH) in surgically and conservatively treated patients, according to potential predictors in Denmark 1996–2011. (DOCX)

S1 Text User definitions of warfarin-platelet inhibitor-, and NSAID therapy. (DOCX)
