# Supplementary material for: Recurrent Intracerebral Hemorrhage: Associations with Comorbidities and Medicine with Antithrombotic Effects
Source: PLoS One. 2016 Nov 10;11(11):e0166223. doi: 10.1371/journal.pone.0166223 (PMC5104445; doi:10.1371/journal.pone.0166223)
Supplement: S1 Table — (DOCX) [file pone.0166223.s001.docx]

**S1 Table**

**Disease diagnoses and types of medicine, according to the International Classification of Diseases (ICD) and the Anatomical Therapeutic Chemical (ATC) Classification System.**

|  | **ICD-10 codes** | **ICD-8 codes** | **ATC codes** |
| --- | --- | --- | --- |
| **Outcome** | | | |
| Intracerebral haemorrhage | I61 | 43100, 43108, 43109, 43190, 43198, 43199 |  |
| **Comorbidities** | | | |
| Atrial fibrillation | I48 | 427.93, 427.94 |  |
| Ischemic heart disease | I20-25 | 410-414 |  |
| Cerebral infarction | I63 | 432-434 |  |
| Biological or mechanical heart valve | KFGE10, KFGE20, KFJF10, KFJF20, KFJF30, KFKD10, KFKD20, FMD10, KFMD11, FMD14, KFMD20  KFGE00, KFJF00, KFKD00, KFMD00 | 308.00, 308.10, 31129, 31268  31130, 31269 |  |
| Antihypertensive treatment  (defined by antihypertensive drugs; ACE-inhibitors, angiotensin receptor blockers, calcium channel blockers, beta-blockers, and diuretics) |  |  | C02 (except C02KX), C03, C07, C08, C09A, C09B, C09C, C09D |
| Diabetes  (defined by anti-diabetic medicine; including both insulin and oral anti-diabetic medicine) |  |  | A10 |
| Alcohol addiction  (defined by diagnosis of alcohol abuse, including intoxication diagnosis, or use of drugs for alcohol dependence) | F10, G31.2, G62.1, G72.1, I42.6, K29.2, K70, K85.2, K86.0, L27.8A, Z72.1 | 303, 57710, 57711, 5711, 29199, 29139, 29129, 29119, 57109 | N07BB |
| Renal insufficiency | N17-N19 | 5932 |  |
| Chronic hepatic diseases | K70-K77 | 571, 573 |  |
| Endocarditis | I38-I39 | 421 |  |
| **Exclusion diagnoses** |  |  |  |
| Intracranial tumor | C70  C71  D32.0  D33.0-2 | 191x, 19219, 19449, 22500-09, 22620-1, 22629, 22639, 22701-2, 23819, 23839 |  |
| Cerebral vascular malformation | Q28.0-3 |  |  |
| Other intracranial bleedings | DI60, DI62,  S06.4-6, S06.8 | 43000, 43001, 43008, 43009, 43090, 43091, 43098, 43099, 43101, 43191, 80300, 80301,  80310, 80311,  80390, 80391, |  |
| Other intracranial bleedings  Continued |  | 85200-02, 85209-12, 85219, 85290-92, 85299, 85309, 85319, 85399, 95859 |  |
| **Medicine** | | | |
| Acetylsalicylic acid |  |  | B01AC06 |
| Clopidogrel |  |  | B01AC04 |
| Warfarin |  |  | B01AA03 |
| NSAID’s |  |  | M01A |
| Statins |  |  | C10AA |
| SSRI’s |  |  | N06AB |
| **Surgical procedures** |  |  |  |
| Removal of spontaneous ICH | KAAB30 |  |  |
| **Exclusion diagnoses for recurrent ICH within 3 months after the primary ICH** | | | |
| Sepsis | DA40-41, DT814D |  |  |
| Pneumonia | DJ12-DJ18, DJ69 |  |  |
| Urinary tract infection | DN30 |  |  |
| Deep venous thrombosis and pulmonary embolism | DI26, DT817C,  DI828-29 |  |  |
| Seizure | DG401, DG402, DG406, DG409, |  |  |
| Unspecified neurological symptoms | DR298A, DZ033, DZ039 |  |  |
