# Supplementary material for: Recurrent Intracerebral Hemorrhage: Associations with Comorbidities and Medicine with Antithrombotic Effects
Source: PLoS One. 2016 Nov 10;11(11):e0166223. doi: 10.1371/journal.pone.0166223 (PMC5104445; doi:10.1371/journal.pone.0166223)
Supplement: S2 Table — (DOCX) [file pone.0166223.s002.docx]

**S2 Table**

**Rate ratio (RR) of recurrent intracerebral hemorrhage (ICH) in surgically and conservatively treated patients, according to potential predictors in Denmark 1996-2011.**

| Potential predictors for recurrent ICH | **Conservative treatment** | | | **Surgical treatment** | | | **P-value^+^** |
| --- | --- | --- | --- | --- | --- | --- | --- |
|  | Events | Adjusted RR** | (95 % CI*) | Events | Adjusted RR** | (95 % CI*) |  |
| **Age (years)** |  |  |  |  |  |  |  |
| 20-49 | 180 | 1 | (Ref.) | 42 | 1 | (Ref.) |  |
| 50-69 | 749 | 1.15 | (0.98-1.36) | 105 | 0.83 | (0.59-1.21) | 0.25^e^ |
| 70- | 951 | 1.14 | (0.97-1.35) | 26 | 0.80 | (0.48-1.30) |  |
| **Sex** |  |  |  |  |  |  |  |
| Males | 1022 | 1.07 | (0.97-1.17) | 100 | 1.10 | (0.81-1.49) | 0.85 |
| Females | 858 | 1 | (Ref.) | 73 | 1 | (Ref.) |  |
| **Antihypertensive treatment** |  |  |  |  |  |  |  |
| Yes | 596 | 0.83 | (0.74-0.92) | 40 | 0.78 | (0.54-1.10) | 0.74 |
| No | 1284 | 1 | (Ref.) | 133 | 1 | (Ref.) |  |
| **Previous cerebral infarction** |  |  |  |  |  |  |  |
| Yes | 245 | 0.89 | (0.77-1.01) | 24 | 1.02 | (0.64-1.53) | 0.56 |
| No | 1635 | 1 | (Ref.) | 149 | 1 | (Ref.) |  |
| **Ischemic heart disease** |  |  |  |  |  |  |  |
| Yes | 258 | 0.93 | (0.81-1.07) | 13 | 0.76 | (0.41-1.29) | 0.49 |
| No | 1622 | 1 | (Ref.) | 160 | 1 | (Ref.) |  |
| **Atrial fibrillation** |  |  |  |  |  |  |  |
| Yes | 168 | 0.82 | (0.69-0.96) | 6 | 0.49 | (0.19-1.02) | 0.19 |
| No | 1712 | 1 | (Ref.) | 167 | 1 | (Ref.) |  |
| **Heart valve surgery** |  |  |  |  |  |  |  |
| Yes | 19 | 1.06 | (0.64-1.65) | 1 | 0.68 | (0.04-3.06) | 0.64 |
| No | 1861 | 1 | (Ref.) | 172 | 1 | (Ref.) |  |
| **Endocarditis** |  |  |  |  |  |  |  |
| Yes | 4 | 1.13 | (0.34-2.69) | 0 | 0.00 | (0.00-5.53) | 0.38 |
| No | 1876 | 1 | (Ref.) | 173 | 1 | (Ref.) |  |
| **Coagulopathy** |  |  |  |  |  |  |  |
| Yes | 12 | 0.78 | (0.41-1.31) | 1 | 0.55 | (0.03-2.45) | 0.72 |
| No | 1868 | 1 | (Ref.) | 172 | 1 | (Ref.) |  |
| **Diabetes mellitus** |  |  |  |  |  |  |  |
| Yes | 73 | 0.99 | (0.77-1.25) | 5 | 1.20 | (0.42-2.63) | 0.70 |
| No | 1807 | 1 | (Ref.) | 168 | 1 | (Ref.) |  |
| **Renal insufficiency** |  |  |  |  |  |  |  |
| Yes | 66 | 1.69 | (1.30-2.15) | 6 | 2.26 | (0.89-4.67) | 0.52 |
| No | 1814 | 1 | (Ref.) | 167 | 1 | (Ref.) |  |
| **Chronic liver disease** |  |  |  |  |  |  |  |
| Yes | 51 | 1.02 | (0.75-1.37) | 6 | 1.14 | (0.44-2.39) | 0.81 |
| No | 1829 | 1 | (Ref.) | 167 | 1 | (Ref.) |  |
| **Alcohol addiction** |  |  |  |  |  |  |  |
| Yes | 157 | 0.90 | (0.75-1.08) | 14 | 0.87 | (0.48-1.45) | 0.88 |
| No | 1723 | 1 | (Ref.) | 159 | 1 | (Ref.) |  |
| **Statins** |  |  |  |  |  |  |  |
| Yes | 224 | 0.99 | (0.85-1.15) | 19 | 1.14 | (0.73-1.92) | 0.43 |
| No | 1656 | 1 | (Ref.) | 154 | 1 | (Ref.) |  |
| **PDMD**^d^ |  |  |  |  |  |  |  |
| Yes | 27 | 1.25 | (0.82-1.80) | 0 | 0.00 | (0.00-3.74) | 0.26 |
| No | 1853 | 1 | (Ref.) | 173 | 1 | (Ref.) |  |

^a^ 95 % Confidence interval

^b^ Rate ratios adjusted for age, gender, calendar period, time since admittance for first ICH, length of hospital stay for the primary ICH, potential predictors listed in Table 1 and current use of antithrombotic treatments, NSAID’s and SSRI’s.

^c^ P-value: test for similar RR’s for surgically vs conservatively treated patients for each predictive factor.

^d^ PDMD: Pre-packaged Daily Medication Doses

^e^ Homogeneity test
