# Supplementary material for: Recurrent Intracerebral Hemorrhage: Associations with Comorbidities and Medicine with Antithrombotic Effects
Source: PLoS One. 2016 Nov 10;11(11):e0166223. doi: 10.1371/journal.pone.0166223 (PMC5104445; doi:10.1371/journal.pone.0166223)
Supplement: S1 Text — (DOCX) [file pone.0166223.s003.docx]

**S1 Text**

**User definition for antithrombotic treatment, NSAID’s, and SSRI’s for the analyses of potential predictors**

To calculate the treatment period for each filled prescription, we assumed that one tablet of acetylsalicylic acid (tablets of 75mg and 150mg) or clopidogrel (tablets of 75mg) corresponded to one day of treatment. For warfarin (tablets of 2,5mg) there is no standard dosage and treatment is regulated for each individual according to INR (international normalized ratio). We assumed a daily dosage of 5 mg. For SSRI’s we calculated with one tablet corresponding to one day of treatment. For each type of NSAID reported to have hemorrhagic side effects, we calculated the daily dosages from the strength of the tablets and the standard dosages recommended for chronic users. For instance, we assumed a daily intake of 1800 mg of ibuprofen, if 600 mg tablets were prescribed.

To avoid overestimation of exposure time, current treatment was always based on the last filled prescription and any prescription was restricted to last for a maximum of 365 days. Patients were moved to the unexposed group when no new prescription was filled after the treatment period of the most recent prescription plus a number of days corresponding to 10% of the treatment time, allowing “slow refillers” to remain in the exposed group. Individuals filling a new prescription of antithrombotic medicine at any time during follow-up reentered the exposed group from the dispensing date. Patients were censored during hospital stays, as medicine use is not recorded here. After discharge, unexposed patients reentered the cohort, and exposed individuals were, to account for any adjustments in treatment, continuously censored until a new prescription was filled.
